# Supplementary material for: German medical students´ views regarding artificial intelligence in medicine: A cross-sectional survey
Source: PLOS Digit Health. 2022 Oct 4;1(10):e0000114. doi: 10.1371/journal.pdig.0000114 (PMC9931368; doi:10.1371/journal.pdig.0000114)
Supplement: S1 Table — (DOCX) [file pdig.0000114.s001.docx]

## **S1 Table. Artificial intelligence knowledge and perceived usefulness in medicine**

| **Question** | **N** | **1 = I do not agree at all - 7 = I completely agree^a^**  **n(%)** | | | | | | | **Median, Mean** | **Inter-quartile range** |
| --- | --- | --- | --- | --- | --- | --- | --- | --- | --- | --- |
|  |  | 1 | 2 | 3 | 4 | 5 | 6 | 7 |  |  |
| Overall, I have good digital skills and competences | 838 | 7  (0.8) | 39  (4.7) | 80 (9.6) | 102 (12.2) | 226 (27) | 279 (33.3) | 105 (12.5) | 5, 5.1 | 2 |
| I feel well informed about AI in medicine | 838 | 99  (11.8) | 252  (30.1) | 188 (22.4) | 137 (16.3) | 107 (12.8) | 40  (4.8) | 15  (1.8) | 3, 3.1 | 2 |
| AI has especially useful applications in the medical field | 807 | 7  (0.9) | 39  (4.8) | 70  (8.7) | 228  (28.3) | 234  (29) | 167  (20.7) | 62  (7.7) | 5, 4.7 | 2 |
| In what area of medicine will AI be particularly useful? | | | | | | | | | | |
| Supporting physicians making a diagnosis | 834 | 31  (3.7) | 77  (9.2) | 105  (12.6) | 99  (11.9) | 231  (27.7) | 207  (24.8) | 84  (10.1) | 5, 4.7 | 3 |
| Supporting physicians in making treatment decisions | 837 | 29  (3.5) | 79  (9.4) | 115  (13.7) | 159  (18.9) | 242  (28.9) | 164  (19.6) | 49  (5.9) | 5, 4.4 | 3 |
| Supporting physicians directly during treatment | 835 | 54  (6.5) | 79  (9.5) | 108  (12.9) | 103  (12.3) | 211  (25.3) | 192  (22.9) | 88  (10.5) | 5, 4.5 | 3 |
| Patients treating themselves independently with AI health apps | 836 | 270  (32.3) | 252  (30.1) | 156  (18.7) | 68  (8.1) | 49  (5.9) | 30  (3.6) | 11  (1.3) | 2, 2.4 | 2 |
| Improvement of drug research and development | 834 | 5  (0.6) | 17  (2.0) | 27  (3.2) | 97  (11.6) | 209  (25.1) | 310  (37.2) | 169  (20.3) | 6, 5.5 | 1 |
| Supporting personalised medicine | 836 | 27  (3.2) | 51  (6.1) | 72  (8.6) | 192  (22.9) | 237  (28.3) | 178  (21.3) | 79  (9.4) | 5, 4.7 | 2 |

^a^ Due to an error uploading the questions to the EvaSys platform, questions in the first section of the survey were put on a 7-point scale.
