# Supplementary material for: German medical students´ views regarding artificial intelligence in medicine: A cross-sectional survey
Source: PLOS Digit Health. 2022 Oct 4;1(10):e0000114. doi: 10.1371/journal.pdig.0000114 (PMC9931368; doi:10.1371/journal.pdig.0000114)
Supplement: S2 Table — (DOCX) [file pdig.0000114.s002.docx]

## **S2 Table. Conflicts between judgement of physician and AI**

| **Question** | **N** | **1 = I do not agree at all - 9 = I completely agree**  **n(%)** | | | | | | | | | **Median, Mean** | **Inter-quartile range** |
| --- | --- | --- | --- | --- | --- | --- | --- | --- | --- | --- | --- | --- |
|  |  | 1 | 2 | 3 | 4 | 5 | 6 | 7 | 8 | 9 |  |  |
| If the physician's judgment and the judgment of a well-tested and accurate AI algorithm differ with regard to a treatment decision, then... | | | | | | | | | | | | |
| ...the judgement of the physician should be followed | 833 | 5  (0.6) | 12  (1.4) | 21  (2.5) | 27  (3.2) | 81 (9.7) | 100 (12) | 191  (22.9) | 228  (27.4) | 168  (20.2) | 7, 7.1 | 2 |
| ...the judgement of the algorithm should be followed | 833 | 147  (17.6) | 211 (25.3) | 170 (20.4) | 126 (15) | 109 (13.1) | 36 (4.3) | 16 (1.9) | 14 (1.7) | 4  (0.5) | 3, 3.1 | 2 |
| ...the patient should choose which judgement should be followed | 823 | 223  (27.1) | 87  (10.6) | 96 (11.7) | 60  (7.3) | 106 (12.9) | 70  (8.5) | 70  (8.5) | 52  (6.3) | 59  (7.2) | 4, 4.0 | 5 |
