# Supplementary material for: German medical students´ views regarding artificial intelligence in medicine: A cross-sectional survey
Source: PLOS Digit Health. 2022 Oct 4;1(10):e0000114. doi: 10.1371/journal.pdig.0000114 (PMC9931368; doi:10.1371/journal.pdig.0000114)
Supplement: S3 Table — (DOCX) [file pdig.0000114.s003.docx]

## **S3 Table. Advantages of AI in medicine**

| **Question** | **N** | **1 = I do not agree at all - 9 = I completely agree**  **n(%)** | | | | | | | | | | | | | | | **Median, Mean** | | | **Inter-quartile range** |
| --- | --- | --- | --- | --- | --- | --- | --- | --- | --- | --- | --- | --- | --- | --- | --- | --- | --- | --- | --- | --- |
|  |  | 1 | 2 | 3 | 4 | | 5 | | 6 | | 7 | | 8 | | 9 | |  |  |  |  |
| I find the following possible advantages of using AI in medicine important | | | | | | | | | | | | | | | | | | | | |
| Analysis of large amounts of clinically relevant data | 832 | 4  (0.5) | 2 (0.2) | 9  (1.1) | | 7  (0.8) | | 16  (1.9) | | 52  (6.3) | | 130  (15.6) | | 236  (28.4) | | 376  (45.2) | | 8, 8.0 | 2 | |
| Making more accurate treatment decisions | 831 | 16  (1.9) | 58  (7.0) | 71  (8.5) | | 69  (8.3) | | 89  (10.7) | | 160  (19.3) | | 190  (22.9) | | 122  (14.7) | | 56  (6.7) | | 6, 5.8 | 3 | |
| Reducing medical errors | 825 | 4  (0.5) | 6  (0.7) | 19  (2.3) | | 21  (2.5) | | 42  (5.1) | | 107  (13) | | 237  (28.8) | | 210  (25.5) | | 179  (21.7) | | 7, 7.2 | 1 | |
| Improving the cost efficiency of medicine | 825 | 30  (3.6) | 35  (4.2) | 47  (5.7) | | 53  (6.4) | | 94  (11.4) | | 103  (12.5) | | 165  (20) | | 159  (19.3) | | 139  (16.8) | | 7, 6.3 | 3 | |
| Giving physicians more time for discussions and clinical  examinations | 823 | 6  (0.7) | 4  (0.5) | 9  (1.1) | | 11  (1.3) | | 29  (3.5) | | 65  (7.9) | | 156  (18.9) | | 250  (30.4) | | 293  (35.8) | | 8, 7.7 | 2 | |
| AI does not get tired and can work 24 hours | 827 | 21  (2.5) | 22  (2.7) | 32  (3.9) | | 38  (4.6) | | 54  (6.5) | | 96  (11.6) | | 135  (16.3) | | 153  (18.5) | | 276  (33.4) | | 8, 7.0 | 3 | |
