# Supplementary material for: German medical students´ views regarding artificial intelligence in medicine: A cross-sectional survey
Source: PLOS Digit Health. 2022 Oct 4;1(10):e0000114. doi: 10.1371/journal.pdig.0000114 (PMC9931368; doi:10.1371/journal.pdig.0000114)
Supplement: S4 Table — (DOCX) [file pdig.0000114.s004.docx]

## **S4 Table. Differences among groups regarding advantages of AI in medicine**

| **Question** | **N** | **1 = I do not agree at all - 9 = I completely agree** | |
| --- | --- | --- | --- |
|  |  | **Gender** | **Digital Skills and Competency** |
| I find the following possible advantages of using AI in medicine important | | | |
| Analysis of large amounts of clinically relevant data | 832 | **Females: 7.9**  **Males: 8.1**  **t(558)=-2.5, *P*=0.01, d=0.21** | **ρ=0.19, *P*<.001** |
| Make more precise treatment decisions | 831 | **Females: 5.6**  **Males: 6.2**  **t(532)=-3.9, *P*<.001, d=0.34** | **ρ=0.08, *P*=.02** |
| Reduction of medical errors | 825 | **Females: 7.076350**  **Males: 7.515267**  **t(623)=-4.1, *P*<.001, d=0.33** | **ρ=0.12, *P*<.001** |
| Improving the cost efficiency of medicine | 825 | Females: 6.4  Males: 6.3  t(454)=0.1, *P*=.89, d<0.01 | ρ=0.05, *P*=.12 |
| Gives physicians more time for discussions and clinical  examinations | 823 | Females: 7.7  Males: 7.7  t(510)=0.03, *P*=.97, d<0.01 | ρ=0.07, *P*=.06 |
| AI does not get tired and can work 24 hours | 827 | **Females: 6.9**  **Males: 7.4**  **t(560)=-3.3, *P*<.001, d=0.28** | ρ=0.03, *P*=.04 |

Note: We used Welch's t-test to compare males and females. This t-test does not assume equal variances for the two samples and adjusts the degrees of freedom accordingly, thereby leading to lower values.
