# Supplementary material for: German medical students´ views regarding artificial intelligence in medicine: A cross-sectional survey
Source: PLOS Digit Health. 2022 Oct 4;1(10):e0000114. doi: 10.1371/journal.pdig.0000114 (PMC9931368; doi:10.1371/journal.pdig.0000114)
Supplement: S5 Table — (DOCX) [file pdig.0000114.s005.docx]

## **S5 Table. Disadvantages of AI in medicine**

| **Question** | **N** | **1 = I do not agree at all - 9 = I completely agree**  **n(%)** | | | | | | | | | **Median, Mean** | **Inter-quartile range** | |
| --- | --- | --- | --- | --- | --- | --- | --- | --- | --- | --- | --- | --- | --- |
|  |  | 1 | 2 | 3 | 4 | 5 | 6 | 7 | 8 | 9 |  |  |  |
| How important do you consider the following possible disadvantages of using artificial intelligence in medicine? | | | | | | | | | | | | | |
| Cannot be used for advice in unforeseen situations  due to insufficient information | 815 | 5  (0.6) | 10  (1.2) | 18  (2.2) | 31  (3.8) | 51  (6.3) | 82  (10.1) | 163  (20) | 241  (29.6) | 214  (26.3) | 8, 7.3 | | 2 |
| Not flexible enough to be used for every patient | 816 | 12  (1.5) | 35  (4.3) | 44  (5.4) | 39  (4.8) | 45  (5.5) | 98  (12) | 137  (16.8) | 204  (25) | 202  (24.8) | 7, 6.9 | | 3 |
| Can amplify biases that already exist in data sets and lead to patient discrimination | 812 | 28  (3.4) | 50  (6.2) | 63  (7.8) | 61  (7.5) | 84  (10.3) | 119  (14.7) | 167  (20.6) | 127  (15.6) | 113  (13.9) | 7, 6.0 | | 3 |
| Can undermine the autonomy of patients | 814 | 23  (2.8) | 49  (6.0) | 83  (10.2) | 82  (10.1) | 83  (10.2) | 119  (14.5) | 150  (18.4) | 130  (15.9) | 95  (11.7) | 6, 5.9 | | 4 |
| Can undermine the autonomy of physicians | 808 | 17  (2.1) | 14  (1.7) | 37  (4.6) | 44  (5.4) | 49  (6.1) | 116  (14.4) | 182  (22.5) | 192  (23.8) | 157  (19.4) | 7, 6.8 | | 2 |
| The lack of ability to develop empathy  and consider the patient´s emotional well-being of the patient | 808 | 7  (0.9) | 13  (1.6) | 22  (2.7) | 10  (1.2) | 21  (2.6) | 20  (2.5) | 105  (12.9) | 201  (24.9) | 409  (50.6) | 9, 7.9 | | 1 |
| Can be developed by programmers with little experience in medical practice | 814 | 21  (2.6) | 22  (2.7) | 57  (7.0) | 45  (5.5) | 66  (8.1) | 86  (10.6) | 154  (18.9) | 178  (21.9) | 185  (22.7) | 7, 6.7 | | 3 |
| Causes uncertainty as to who is liable in the event of an error | 813 | 19  (2.3) | 17  (2.1) | 29  (3.6) | 23  (2.8) | 59  (7.3) | 80  (9.8) | 175  (21.5) | 176  (21.6) | 235  (28.9) | 8, 7.1 | | 3 |
