# Supplementary material for: German medical students´ views regarding artificial intelligence in medicine: A cross-sectional survey
Source: PLOS Digit Health. 2022 Oct 4;1(10):e0000114. doi: 10.1371/journal.pdig.0000114 (PMC9931368; doi:10.1371/journal.pdig.0000114)
Supplement: S6 Table — (DOCX) [file pdig.0000114.s006.docx]

## **S6 Table. Differences among groups regarding disadvantages of AI in medicine**

| **Question** | **N** | **1 = I do not agree at all - 9 = I completely agree** | |
| --- | --- | --- | --- |
|  |  | **Gender** | **Digital skills and competency** |
| How important do you consider the following possible disadvantages of using artificial intelligence in medicine? | | | |
| Cannot be used for advice in unforeseen situations  due to insufficient information | 815 | **Females: 7.4**  **Males: 7.1**  **t(430)=2.6, *P*=.008, d=0.25** | ρ=0.02, *P*=.60 |
| Not flexible enough to be used for every patient | 816 | **Females: 7.2**  **Males: 6.0**  **t(409)=7.1, *P*<.001, d=0.70** | ρ=-0.06, *P*=.09 |
| Can amplify biases that already exist in data sets and use them to discriminate against patients | 812 | Females: 6.1  Males: 5.8  t(487)=1.6, *P*=.1, d=0.15 | ρ=-0.04, *P*=.21 |
| Can undermine the autonomy of patients | 814 | **Female: 6.0**  **Males: 5.5**  **t(465)=2.7, *P*=.006, d=0.25** | **ρ=-0.12, *P*<.001** |
| Can undermine the autonomy of physicians | 808 | **Females: 6.9**  **Males:6.4**  **t(437)=3.3, *P*=.001, d=0.32** | ρ=-0.06, *P*=.09 |
| The lack of ability to develop empathy  and to take into account the emotional well-being of the patient | 808 | **Females: 8.1**  **Males: 7.6**  **t(407)=3.7, *P*<.001, d=0.37** | ρ=-0.05, *P*=.14 |
| Can be developed by programmers with little experience in medical practice | 814 | Females: 6.7  Males: 6.6  t(476)=0.6, *P*=.06**, d=0.1** | ρ=0.01, *P*=.75 |
| Causes uncertainty as to who is liable in the event of an error | 813 | **Females: 7.2**  **Males: 6.8**  **t(436)=2.5, *P*=.01, d=0.24** | ρ=0.02, *P*=.65 |

Note: We used Welch's t-test to compare males and females. This t-test does not assume equal variances for the two samples and adjusts the degrees of freedom accordingly, thereby leading to lower values.
