# Supplementary material for: German medical students´ views regarding artificial intelligence in medicine: A cross-sectional survey
Source: PLOS Digit Health. 2022 Oct 4;1(10):e0000114. doi: 10.1371/journal.pdig.0000114 (PMC9931368; doi:10.1371/journal.pdig.0000114)
Supplement: S7 Table — (DOCX) [file pdig.0000114.s007.docx]

## **S7 Table. Implementation issues**

| **Question** | **N** | **1 = I do not agree at all - 9 = I completely agree**  **n(%)** | | | | | | | | | | | | | | | **Median, Mean** | | **Inter-quartile range** | |
| --- | --- | --- | --- | --- | --- | --- | --- | --- | --- | --- | --- | --- | --- | --- | --- | --- | --- | --- | --- | --- |
|  |  | 1 | 2 | 3 | 4 | | 5 | | | 6 | 7 | | 8 | | 9 | |  |  |  |  |
| When AI is used in medicine, it is important... | | | | | | | | | | | | | | | | | | | | |
| That the underlying data of the algorithms are representative | 801 | 1  (0.1) | 3  (0.4) | 8  (1.0) | | 4  (0.5) | | 33  (4.1) | 55  (6.9) | | | 132  (16.5) | | 222  (27.7) | | 343  (42.9) | | 8, 7.9 | | 2 |
| That the developers can explain the rules and parameters of the algorithm to physicians | 803 | 1  (0.1) | 1  (0.1) | 5  (0.6) | | 8  (1.0) | | 20  (2.5) | 32  (3.9) | | | 110  (13.7) | | 193  (24) | | 433  (53.9) | | 9, 8.2 | | 1 |
| That physicians were consulted before introducing the AI algorithm system in clinical practice | 802 | 0 | 1  (0.1) | 1  (0.1) | | 2  (0.2) | | 22  (2.7) | 28  (3.5) | | | 107  (13.3) | | 175  (21.8) | | 466  (58.1) | | 9, 8.3 | | 1 |
| That physicians have a choice whether to use an AI algorithm | 790 | 2  (0.3) | 11  (1.4) | 29  (3.7) | | 48  (6.1) | | 54  (6.8) | 80  (10.1) | | | 158  (20) | | 156  (19.7) | | 252  (31.9) | | 8, 7.2 | | 3 |
| That patients are always informed when AI algorithms are used | 788 | 3  (0.4) | 6  (0.8) | 17  (2.2) | | 10  (1.3) | | 15  (1.9) | 30  (3.8) | | | 77  (9.8) | | 133  (16.9) | | 497  (63.1) | | 9, 8.2 | | 1 |
| That patients have a choice whether AI algorithms are used in their treatment | 782 | 15  (1.9) | 22  (2.8) | 31  (3.9) | | 32  (4.1) | | 42  (5.4) | 64  (8.2) | | | 111  (14.2) | | 127  (16.2) | | 338  (43.2) | | 8, 7.3 | | 3 |
| That patients have equal access to the system | 779 | 44  (5.6) | 58  (7.3) | 120  (15.4) | | 58  (7.3) | | 105  (13.5) | 71  (9.1) | | | 73  (9.4) | | 72  (9.2) | | 178  (22.8) | | 6, 5.6 | | 5 |
| That oversight mechanisms are in place to evaluate the performance of an AI algorithm in clinical practice | 775 | 3  (0.4) | 4  (0.5) | 10 (1.3) | | 11  (1.4) | | 21  (2.7) | 33  (4.3) | | | 112  (14.5) | | 170  (21.9) | | 411  (53) | | 9, 8.0 | | 1 |
| That there are legal rules to clarify liability in case of an error | 776 | 2  (0.3) | 3  (0.4) | 3  (0.4) | | 5  (0.6) | | 10  (1.3) | 19  (2.5) | | | 77  (9.9) | | 163  (21) | | 494  (63.7) | | 9, 8.4 | | 1 |
| The topic of AI should receive a lot of attention in medical studies. | 776 | 10  (1.3) | 21  (2.7) | 63  (8.1) | | 60  (7.7) | | 108  (13.9) | 158  (20.4) | | | 164  (21.1) | | 113  (14.6) | | 79  (10.2) | | 6, 6.1 | | 3 |
