# Supplementary material for: German medical students´ views regarding artificial intelligence in medicine: A cross-sectional survey
Source: PLOS Digit Health. 2022 Oct 4;1(10):e0000114. doi: 10.1371/journal.pdig.0000114 (PMC9931368; doi:10.1371/journal.pdig.0000114)
Supplement: S1 Appendix — (DOCX) [file pdig.0000114.s008.docx]

Institute for History and Ethics of Medicine


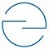

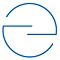


| EvaSys | First Semester Survey WS19/20 [Copy] | 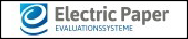 |
| --- | --- | --- |
| Institute for History and Ethics of Medicine | | |

Please mark it so:

Correction:

Please use a ballpoint pen or a felt-tip pen that is not too strong. This questionnaire will be recorded by machine.

In the interest of optimal data collection, please follow the instructions given on the left when completing the form.

1. information on the study

As future physicians, you will regularly encounter moral dilemmas in clinical practice and have to make decisions in these situations. The planned study investigates whether students already perceive ethically challenging situations during their studies, how their moral attentiveness and competence is, and how students reflect on such ethical conflicts. In a further part of the study, ethical aspects of artificially intelligent algorithms for medical diagnostics and therapy will be investigated.

Please mark it so:

Correction:

Please use a ballpoint pen or a felt-tip pen that is not too strong. This questionnaire will be recorded by machine.

In the interest of optimal data collection, please follow the instructions given on the left when completing the form.

In addition, questions are asked about self-reflexivity and insecurity, as well as various attitudes and attitudes relating to the medical profession.

## Aim of the study

The aim of this study is to investigate the general and specific ethical mindfulness and reflective competence of medical students at the beginning of their studies.

## How does the study work?

In the context of the internship of the career exploration, we invite you and about 900 other medical students to voluntarily participate in this study. Please answer the following questionnaire spontaneously, without much thought. There are no wrong answers.

Participation in the study is not associated with any risks other than the usual data protection risks, which are low in this case. The burden is kept within narrow limits - it only takes about 20 minutes to complete the questionnaire. Your participation in the study is independent of the successful completion of the internship; the issuance of the certificate "Internship of Vocational Field Exploration" is done separately. If you do not wish to participate in the study, this will not have any negative consequences for you.

If you are willing to participate in the study, please fill out the questionnaire and the declaration of study participation. Please do not forget to sign the declaration.

If you do not wish to participate in the study, please complete the "Declaration of participation". Then please tick the box "No, I do not wish to participate" in the appropriate place.

participate". Please also enter the reasons for your refusal and answer two short questions about yourself. This information will be treated completely anonymously, i.e. without reference to your name. It is very important for the assessment of our study results and their representativeness. The data are not included in the actual evaluation.

# 2. data protection

## What happens to your data?

In this study, Prof. Dr. Peter Henningsen, Dean of the Medical Faculty, Ismaninger Straße 22, 81675 Munich, secretary's office: [kircher@tum.de,](mailto:kircher@tum.de) is responsible for data processing. The processing of your data requires your consent (legal basis). Your data will be used exclusively in the context of this study. This includes personal identifying data such as age and gender. All data directly identifying your person will be replaced by an identification code (pseudonymised). This largely excludes an identification of your person by unauthorized persons.

Your data will be stored on servers of the Leibniz Computing Center of the Bavarian Academy of Sciences. They will be deleted after 10 years. The consent to the processing of your data is voluntary, you can revoke the consent at any time without giving reasons and without disadvantages for you. You have the right to obtain information about the data concerning you, also in the form of a free copy. In addition, you can request the correction or deletion of your data.

In these cases, please contact Prof. Dr. Peter Henningsen, Dean of the Medical Faculty, Ismaninger Straße 22, 81675 Munich, secretary's office: [kircher@tum.de.](mailto:kircher@tum.de)

In the event of a complaint, contact: Data Protection Officer

Klinikum rechts der Isar of the Technical University of Munich Ismaninger Str. 22

81675 Munich

Email: [datenschutz@mri.tum.de](mailto:datenschutz@mri.tum.de)

or to:

Bavarian State Commissioner for Data Protection Postal address: Postfach 22 12 19, 80502 Munich, Germany

Home address: Wagmüllerstr. 18, 80538 Munich E-mail: [poststelle@datenschutz-bayern.de.](mailto:poststelle@datenschutz-bayern.de)

Data Protection Officer of the TU Munich E-mail: [beauftragter@datenschutz.tum.de](mailto:beauftragter@datenschutz.tum.de) Technische Universität München

Arcisstr. 21

80333 Munich

# 3. declaration of participation in the study

3.1 Please enter here whether you would like to participate in the survey as part of our study.

Yes, I would like to participate in the study.

No, I do not wish to participate in the study.

3.2 If you do not wish to participate in the study: A short justification would be very helpful for us

If you do not wish to participate, please provide us with two pieces of information about yourself. These are very important for the assessment of our study results and their representativeness. Of course, this data will be treated completely anonymously.

3.3 Your year of birth

3.4 Your gender

female

Male

different

…..

Please read the following statements and indicate on the scale to what extent you agree or disagree with the statements. There are no right or wrong answers.

6. Artificial Intelligence (AI)

6.1 Overall, I have good overall digital skills and competences.

I do not agree at all

I agree completely

6.2 I feel well informed about artificial intelligence (AI) in medicine.

- 1. AI has especially useful applications in the medical field

In which area of medicine will AI be particularly useful?

(Please estimate for each example)

- 1. Supporting physicians in making a diagnosis, for example, via a diagnosis algorithm
  2. Supporting physicians in making treatment decisions, for example, via an algorithm suggesting treatment options
  3. Supporting doctors directly in treatment, for example, via AI robots
  4. Patients treating themselves independently with AI health apps
  5. Improving drug research and development via AI algorithms
  6. Supporting personalised medicine through AI algorithms

I do not agree at all

I agree completely

# 6. Artificial Intelligence

If the physician's judgment and the judgment of a well-test and precise AI algorithm differ regarding a treatment decision, then ...

- 1. ..the judgment of the physician should be followed
  2. ...the judgment of the algorithm should be followed...

6.12 ...the patient should chooses which judgment should be followed

I find the following potential advantages of using AI in medicine important.

6.13 Analysis of large amounts of clinically relevant data

6.14 Making more accurate treatment decisions

6.15 Reducing medical errors

6.16 Improving the cost-effectiveness of medicine

6.17 Giving doctors more time discussions and clinical examinations.

6.18 AI does not get tired and can work 24h

- 1. Others..

How important do you consider the following possible disadvantages of using artificial intelligence in medicine?

- 1. Cannot be used for advice in unforeseen situations

due to insufficient information.

- 1. Not flexible enough to be used for every patient.
  2. Can amplify biases that already exist in

data sets and lead to patient discrimination.

- 1. Can undermine the autonomy of patients

6.24 Can undermine the autonomy of physicians.

- 1. The lack of ability to develop empathy and consider the patient's emotional well-being.
  2. Can be developed by programmers with little

experience in medical practice

- 1. Causes uncertainty about who is liable if something goes wrong.

6.28 Others…

When AI is used to medicine, it's important...

- 1. That the underlying data of the algorithms are representative.
  2. That the developers can explain the rules and

parameters of the algorithm to physcians.

- 1. That physicians were consulted before introducing an AI algorithm system into clinical practice.

6. artificial intelligence (AI)[continued]

- 1. That physicians have a choice whether to use an AI algorithm
  2. That patients are always informed when AI algorithms are used
  3. That patients have a choice whether

AI algorithms are used in their treatment.

- 1. That patients have equal access to the system
  2. That oversight mechanisms are in place

to evaluate the performance of an AI algorithm in clinical practice.

- 1. That there are legal rules to clarify liability in the event of an error

6.38 The topic of AI should receive a lot of

Attention in medical studies.
