# Supplementary material for: German medical students´ views regarding artificial intelligence in medicine: A cross-sectional survey
Source: PLOS Digit Health. 2022 Oct 4;1(10):e0000114. doi: 10.1371/journal.pdig.0000114 (PMC9931368; doi:10.1371/journal.pdig.0000114)
Supplement: Attachment — Submitted filename: PLOS Digital Health Response to Reviewers.docx [file pdig.0000114.s009.docx]

PLOS Digital Health Response to Reviewers

Journal Requirements:

1. Please amend your online Financial Disclosure statement. If you did not receive any funding for this study, please simply state: “The authors received no specific funding for this work.”

2. Please update your online Competing Interests statement. If you have no competing interests to declare, please state: “The authors have declared that no competing interests exist.”

3. Please provide a complete Data Availability Statement in the submission form, ensuring you include all necessary access information or a reason for why you are unable to make your data freely accessible. If your research concerns only data provided within your submission, please write “All data are in the manuscript and/or supporting information files.” as your Data Availability Statement.

Additional Editor Comments (if provided):


Reviewers' comments:

Reviewer #1: Please indicate the original language of the survey. And if the survey was originally in German, please briefly describe how it was translated to English.

Response: Thank you for pointing out this omission. The survey was conducted in German. The survey has been translated into English using back translation. This has been added to the methods.

Was the pilot test done separately? Were the results of the pilot test included in the present study? Were there any changes to the survey due to the pilot testing?

Response: The pilot test was done separately and the results were not included in the present study. No changes to the survey were required. This has been added to the methods.

Some info on whether the Berufsfelderkundung course was related to AI or medical technology or not seems relevant to interpreting the results of this study and thus should be added to the manuscript.

Response: The course provides a career exploration for students and an overview of the fields of medicine; there is no specific focus on AI. This has been added to the methods.

The details of the scales deserve more attention than just being table footnotes. The suggestion here is that the scales could be better off described in the texts preceding the tables, and that it be made clear to the reader what the labels were and that the scales were labeled only on either end.

Response: Thank you for your suggestion, however, we do not think that any changes are required. Details of the scales are provided in the methods section, in the footnotes of the tables in the manuscript, and the full tables and the survey are in the appendix.

The term “middle category” was used in the paper without a clear definition. From the surrounding texts, it seems this meant the central part of the Likert scale. But then a clear definition is still needed, as, on a 9-point Likert scale with labels only on either end, the central part could be defined in multiple ways. Two common examples would be defining only 5 as the “middle category,” and defining 4 to 6 as the “middle category.” Also, some of the questions were done using a 7-point Likert scale, which might require a separate definition of what the “middle category” was. It can be helpful to the reader to clearly describe which points were considered “disagree,” “neutral,” and “agree.” Please clarify.

Response: Thank you for pointing this out, we agree that this needs to be clarified. We have decided to omit reference to the middle neutral category. When reporting absolute and relative frequencies, the majority proportion of participants who agreed or disagreed with the statement is reported; the remaining participants were either neutral (4 in the case of 7-point and 5 in the case of 9-point scale) or agreed/disagreed. We have clarified this in the methods. The tables in the manuscript report median and mean, and the supplement tables report full results.

Judging from the statistical parameters used in presenting the data, the responses were likely non-normally distributed. Still, it would be helpful to clearly indicate the normality of the data to the reader.

Response: The distribution of the items regarding AI were certainly not normally distributed nor did they have to be. When reporting proportions in tables 1–5 we did not set up a statistical model that requires assumptions such as normality. This is precisely why we report median in addition to means in tables 1–5.

Results from Likert scales are poorly parsable when presented in tables, especially without a frequency distribution table, and even with one it still may not help much. Please consider using another form of visual representation. As an example, the following link shows some possible ways to effectively visualize results from Likert scales. <https://www.storytellingwithdata.com/blog/2012/03/visualizing-survey-data>

Response: We thank the reviewer for this suggestion, which we think it generally a good idea. However, since we dichotomized the data when presenting them in the text, a visualization would not really make sense because the dichotomized data would no longer correspond with those plotted in the figures, which would render the manuscript less readable. The figures recommended by the reviewer would only be helpful if one did not dichotomize the data.

As written by the authors, the gender differences seemed to be important findings. Clearly illustrating these differences, whether by a table or a graphic, could help in conveying the importance to the reader. Maybe there can also be further discussions regarding these differences, as there is a clear disparity between how often this topic was mentioned in the Results section and how little attention was given to it in the Discussion section.

Response: Thank you for this point. However, we believe this is overstating things. In the results section we note the in relation to participants´ views about the advantages and disadvantages of medical AI that:

“However, there were some significant differences between genders and participants´ perceived digital skills and competences (Table S4). Male participants were significantly more likely to agree that possible advantages of medical AI include analysis of large amounts of clinically relevant data (t(558)=–2.5, P=0.01, d=0.21), making more precise treatment decisions (t(532)= –3.9, P<.001, d=0.34), the reduction of medical errors (t(623)= –4.1, P<.001, d=0.33), and that AI does not get tired and can work 24 hours (t(560)= –3.3, P<.001, d=0.28). Effect sizes for these gender differences varied between small and small to medium (0.21–0.34).

However, there were some significant differences between genders and participants´ perceived digital skills and competences (Table S6). Female participants were significantly more likely to agree that possible disadvantages of medical AI include that it cannot be used for advice in unforeseen situations due to insufficient information (t(430)=2.6, P=.008, d=0.25), that it is not flexible enough to be used with every patient (t(409)=7.1, P<.001, d=0.70), that it can undermine the autonomy of patients (t(465)=2.7, P=.006, d=0.25), that it can undermine the autonomy of physicians (t(437)=3.3, P=.001, d=0.32), the lack of ability to develop empathy and to take into account the emotional well-being of the patient (t(407)=3.7, P<.001, d=0.37), and that it can cause uncertainty as to who is liable in the event of an error (t(436)=2.5, P=.01, d=0.24). Effect sizes for these gender differences varied between small and small to medium (0.24–0.37), except for flexibility of use which was medium to large (0.70).”

No where else in the results section do we report gender differences. Although there were some gender differences, given the effect sizes were generally small, we do not think it is justified to make much of these differences in the discussion section.

The results showed that most of the participants believed the physician should be trusted over well-calibrated AI in a case of conflict. In general, well-made algorithms are more precise and produce less noise than human judgment. It is thus theoretically better overall to follow the AI’s suggestions. But in the real world, there can be medico-legal reasons to lean on the physician’s side. This part seems to warrant further discussion and carries real teaching points for medical students.

Response: Thank you for your comment. We agree that this is an interesting point. However, although there has been some research that indicates that algorithms can potentially be more accurate than humans in certain cases, there is generally a lack of good evidence on the effectiveness of medical AI and we do not think this can be said generally of medical AI. Nevertheless, we agree that there is a challenge to determine when well tested and accurate tools should be followed and if constraints should be put on clinicians´ behaviour. A similar issue can be seen in the context of evidence-based medicine, which we discuss in the following section of the discussion:

“Students also strongly agreed that the judgement of the physician should be followed in cases of conflict with AI algorithms, that patients are always informed when AI algorithms are used, and that physicians and patients should have a choice whether AI in used. These views fit nicely with current discourse on the dangers of AI confusing responsibility and undermining patients´ and physicians’ ability to make autonomous informed decisions about treatment [16-21,45]. Authorities are still grappling with how to regulate and govern AI technology, and there remains limited legal rules and oversight. It is important that this situation is addressed promptly; future clinicians should not be faced with a workplace where these important issues around responsibility are not clearly regulated. This will include not only how responsibility is allocated when things go wrong, but also how to best respect clinician judgement in learning health care systems using AI technology [46]. The general discussion around AI in medicine has shifted away from complete replacement of physicians and their judgement, to more synergistic uses of AI [31]. Nevertheless, although clinician judgement is indispensable to the practice of medicine, evidence-based medicine movement has shown that intuition and unsystematic clinical experience can be biased, up to 10 years behind latest research, and recommend ineffective therapies [46]. Not all constraints on the behaviour of clinicians will necessarily interfere with the obligation to respect clinicians’ judgement, but contextual assessments of the likely impact of any proposed constraints are required [47].”

The tables represent participants’ opinions on various issues, not those issues themselves. Table names should reflect this.

Response: Thank you, but we think it is sufficiently clear that this study is presenting the participants´ views on these topics. We think adding “students views about…” or similar to each table title is unnecessary.

The fact that the participants had had no experience in medicine (they were fresh out of high school) should also be listed as a limitation of the study. In addition, “high school graduates” is probably a better description of their state than “after school.”

Response: Upon reflecting on this issue, we think that neither “after school” or the suggested “high school graduates” is the correct term to use. Although many students may be coming directly from high school, this is not always the case and new medical students can be older and have studied or worked in other fields before starting medical school. We therefore changed the sentence to read “Although the answers of the students participating in this study do not represent a “medical student” state…”. We note throughout the manuscript, including the limitations section, that participants were “new medical students”.

A space between an equal sign and a minus sign would help with legibility. This might only be a font issue and may not show up in the final manuscript, but it seems prudent to keep this in mind.

Response: A space has been added to the results section between any equal sign followed by a minus sign.

Overall, the manuscript needs to be copyedited for grammar and clarity, particularly on verb conjugation and missing articles/prepositions/punctuations. A few examples are described below.
- On page 5, the sentence starting with “In 2019,...” could use a comma between “radiologists” and “and of the…” to improve clarity. (If I’m understanding it correctly; that is quite a run-on sentence.)
- Page 10, “and” should be added before the final clause of the first and second sentences.
- Page 23, last paragraph, forewent should have been foregone.

Response: Thank you for pointing out these minor errors, they have now been corrected.

Reviewer #2: Information content:
The manuscript describes new research that aimed to better understand medical students knowledge and views regarding the use of AI technology in medicine. It appears that this is one of the first studies to explore medical students´ general views about AI in medicine

There is a high response rate to their survey and the results of the study found relatively low awareness among medical students regarding AI in medicine and the need for curricula to ensure that clinicians are able to fully realize the potential of AI technology.

Introduction:
This section provided an overview of the relevant literature and outlined the key research questions to be addressed.

Response: Thank you.

Methods:
This section has been placed at the back of the paper after the discussion. I recommend it is brought forward following the 'Introduction' section ie in the IMRaD format.

Response: We would also prefer a IMRaD format, but were following journal guidelines that require the section to be placed after the discussion <https://journals.plos.org/digitalhealth/s/submission-guidelines#loc-manuscript-organization>. However, we have rechecked journal guidelines and now see they now allow some flexibility. We have therefore moved the methods section up as suggested.

As stated it's unfortunate that "Due to an error uploading the questions to the EvaSys platform, questions in the first section of the survey were put on a 7-point scale: 1 = I do not agree at all - 7 = I completely agree"
This should be stated in the 'limitations' section. In looking at your survey, it's unclear if student respondents would have realised the scale changed from a 7 point scale to a 9 point scale. Could you add further detail explaining whether you believe this has significantly impacted your results or not?

Response: Thank you for pointing out this oversight. We have now added this to the limitations section. Regarding the question “Could you add further detail explaining whether you believe this has significantly impacted your results or not?”: This is difficult as we don´t really have a way of finding this out. The problem lies in the fact that all participants responded to the items in the first section and not part of them. Thus there is no way of knowing what the results would have been had the scale consisted of a 9- instead of a 7-point Likert scale.

The study aims to assess medical students knowledge of AI - but in the questionnaire there appears to be only two questions:
        1. "Overall, I have good overall digital skills and competences"
        2. I feel well informed about artificial intelligence (AI) in medicine.

It appears the vast majority of the survey is assessing student awareness, attitudes and perceptions of AI rather than explicitly assessing knowledge of AI. This could be further acknowledged in the limitations section.

Response: Yes, this is a good point. It is true that there were not many questions about student´s knowledge of AI in the survey. We have decided to remove “knowledge” from the title and abstract etc to reflect that the focus is on student´s views of AI.

Results and discussion:
The results are clearly outlined in sufficient detail and appropriate tables have been used. The results have been discussed in relation to the relevant literature.

Response: Thank you.

Limitations of the research have been acknowledged - further limitations could be added as outlined above. Future research could also consider qualitative studies to further explore and elaborate on this study's findings. It would be also interesting to know if there are differences by year level of medical school and whether results may change longitudinally over time.

Response: Thank you. We have added to the further limitations as suggested. We agree that such further research would be helpful and have also noted this in the manuscript.
